# Beneficial Effects of Soluble Guanylyl Cyclase Stimulation and Activation in Sickle Cell Disease Are Amplified by Hydroxyurea: In Vitro and In Vivo Studies[Fn FN5]

**DOI:** 10.1124/jpet.119.264606

**Published:** 2020-09

**Authors:** W.A. Ferreira, H. Chweih, C. Lanaro, C.B. Almeida, P.L. Brito, E.M.F. Gotardo, L. Torres, L.I. Miguel, C.F. Franco-Penteado, F.C. Leonardo, F. Garcia, S.T.O. Saad, P.S. Frenette, D. Brockschnieder, F.F. Costa, J.P. Stasch, P. Sandner, N. Conran

**Affiliations:** Hematology Center, School of Medical Sciences, University of Campinas (UNICAMP), Brazil (W.A.F., H.C., C.L., C.B.A., P.L.B., E.M.F.G., L.T., L.I.M., C.F.F.-P., F.C.L., F.G., S.S.T.O., F.F.C., N.C.); Bayer AG, Pharmaceuticals - Drug Discovery, Wuppertal, Germany (D.B., J.P.S., P.S.); Ruth L. and David S Gottesman Institute for Stem Cell and Regenerative Medicine Research, Albert Einstein College of Medicine, Bronx, New York (P.S.F.); and Hannover Medical School, Institute of Pharmacology, Hannover, Germany (P.S.)

## Abstract

The complex pathophysiology of sickle cell anemia (SCA) involves intravascular hemolytic processes and recurrent vaso-occlusion, driven by chronic vascular inflammation, which result in the disease’s severe clinical complications, including recurrent painful vaso-occlusive episodes. Hydroxyurea, the only drug frequently used for SCA therapy, is a cytostatic agent, although it appears to exert nitric oxide/soluble guanylyl cyclase (sGC) modulating activity. As new drugs that can complement or replace the use of hydroxyurea are sought to further reduce vaso-occlusive episode frequency in SCA, we investigated the effects of the sGC agonists BAY 60-2770 (sGC activator) and BAY 41-2272 (sGC stimulator) in the presence or absence of hydroxyurea on SCA vaso-occlusive mechanisms and cell recruitment both ex vivo and in vivo. These agents significantly reduced stimulated human SCA neutrophil adhesive properties ex vivo in association with the inhibition of surface *β*2-integrin activation. A single administration of BAY 60-2770 or BAY 41-2272 decreased tumor necrosis factor cytokine–induced leukocyte recruitment in a mouse model of SCA vaso-occlusion. Importantly, the in vivo actions of both agonists were significantly potentiated by the coadministration of hydroxyurea. Erythroid cell fetal hemoglobin (HbF) elevation is also a major goal for SCA therapy. BAY 41-2272 but not BAY 60-2770 at the concentrations employed significantly induced *γ*-globin gene transcription in association with HbF production in cultured erythroleukemic cells. In conclusion, sGC agonist drugs could represent a promising approach as therapy for SCA, for use either as stand-alone treatments or in combination with hydroxyurea.

**SIGNIFICANCE STATEMENT:**

This preclinical study demonstrates that stimulators and activators of sGC are potent inhibitors of the adhesion and recruitment of leukocytes from humans and in mice with sickle cell anemia (SCA) and may represent a promising approach for diminishing vaso-occlusive episode frequency in SCA. Hydroxyurea, a drug already frequently used for treating SCA, was found to potentiate the beneficial effects of sGC agonists in in vivo studies, implying that these classes of compounds could be used alone or in combination therapy.

## Introduction

SCA, an inherited disease, is caused by the generation of abnormal sickle hemoglobin, which polymerizes at low oxygen levels, making the red blood cell more rigid and sickle shaped (Eaton and Hofrichter, 1987). SCA incurs numerous clinical complications, including frequent painful vaso-occlusive episodes that often require hospitalization, acute chest syndrome, stroke, renal damage, pulmonary hypertension, and a shortened lifespan (Steinberg, 2009). Sickle hemoglobin polymerization promotes a vast range of pathophysiological alterations, including changes in red blood cell function, extra- and intravascular hemolysis, chronic inflammation, and consequently, vaso-occlusion (Conran and Belcher, 2018). Vaso-occlusive processes (responsible for much of the morbidity of SCA) occur principally in the microcirculation (Steinberg, 2016) and are triggered by the interactions of red blood cells, endothelial cells, activated leukocytes, platelets, and plasma proteins via a mechanism in which inflammation, hypoxic events, oxidative stress, and reduced nitric oxide (NO) availability play driving roles (Zhang et al., 2016). In particular, in vivo studies employing mice with sickle cell disease (SCD mice) suggest that the recruitment of activated leukocytes to blood vessel walls is crucial for initiating these multicellular interactions (Turhan et al., 2002; Hidalgo et al., 2009).

Hydroxyurea (or hydroxycarbamide) is one of the few therapeutic options currently employed for reducing the frequency of vaso-occlusive episodes in patients with SCA. Augmentation of fetal hemoglobin (HbF) production in patients with SCA, even at low levels, can decrease sickle hemoglobin polymerization and significantly improve the disease’s clinical course. Hydroxyurea, a cytostatic agent, induces HbF production in erythroid lineage cells, thus diminishing sickling events (Charache et al., 1995). Mounting data also indicate that hydroxyurea acts as an NO donor compound, boosting cGMP levels in vivo, and can react with heme proteins to generate NO (Pacelli et al., 1996; Glover et al., 1999). Additionally, numerous agents are currently in various stages of clinical development for SCA therapy, many of which have been identified using a pathophysiology-based approach that targets at least one of the mechanisms that contributes to the pathology of the disease (Conran and Belcher, 2018).

Reduced bioavailability of NO, which is principally due to the consumption of vascular NO by cell-free hemoglobin during hemolysis, may contribute to SCA vaso-occlusive processes (Reiter et al., 2002). As the consumption of NO after acute hemolytic processes leads to rapid inflammatory responses that result in substantial leukocyte recruitment to the blood vessel wall (Almeida et al., 2015), stimulation of sGC (the enzyme target of NO) and intracellular cGMP elevation could represent an approach to reducing inflammation, leukocyte recruitment, and consequent vaso-occlusive processes in SCA. Indeed, amplification of NO-dependent signaling using inhibitors of phosphodiesterase-9 significantly diminishes vaso-occlusive processes in SCD mice (Almeida et al., 2012; McArthur et al., 2020), and the amelioration of NO bioavailability improves microvascular functions, increases survival, and prevents lung injury during hypoxia in SCD mice (de Franceschi et al., 2003; Kaul et al., 2008).

The sGC enzyme functions normally in its reduced (ferrous) oxidation state; however, oxidative conditions can make the sGC heme unresponsive to NO or even result in its loss from the enzyme (Stasch et al., 2006). Depending on its oxidative state, the enzyme can be pharmacologically stimulated by either sGC stimulators or sGC activators. BAY 41-2272 is a prototype compound of a class of NO-independent sGC stimulators; this molecule binds to a regulatory site on the *α*-subunit of sGC and stimulates the native nonoxidized enzyme without NO but also synergistically with NO. BAY 41-2272 can inhibit platelet aggregation in vitro and leukocyte adhesion in vivo (Hobbs and Moncada, 2003; Ahluwalia et al., 2004). On the other hand, for stimulation of the oxidized sGC, in which there is the loss of function of the heme group, heme-independent sGC activators are required (Stasch et al., 2006). BAY 60-2770 is an NO- and heme-independent sGC activator (Pankey et al., 2011) that, given the oxidative stress associated with SCD (van Beers and van Wijk, 2018), could be of therapeutic benefit in this disease.

It is conceivable that a multidrug approach to treating SCA will evolve in the coming years in which drugs can be used alone or in combination with others, such as hydroxyurea, to amplify NO-dependent signaling and diminish inflammation (Conran and Torres, 2019), among other mechanisms. The aim of this study was to investigate the effects of BAY 60-2770 (sGC activator) and of BAY 41-2272 (sGC stimulator), administered in the absence or presence of hydroxyurea, on the inflammatory mechanisms that contribute to SCA vaso-occlusive processes in vitro and in vivo. In addition, modulation of HbF production in erythroid cells by these agents was evaluated.

## Materials and Methods

### Reagents

BAY 60-2770 and BAY 41-2272 were provided by Bayer AG (Wuppertal, Germany). 1H-[1,2,4]oxadiazolo[4,3-a]quinoxalin-1-one (ODQ) was purchased from Cayman Chemical (Ann Arbor, MI), and hemin was obtained from Frontier Scientific (Newark, NJ). Recombinant human and murine tumor necrosis factor-*α* (TNF) and human fibronectin were from R&D Systems (Minneapolis, MN). Dulbecco’s modified Eagle’s medium, fetal bovine serum, penicillin, and streptomycin were from Gibco-Invitrogen (New York, NY). Trizol was from Invitrogen (Carlsbad, CA). All other reagents were from Sigma-Aldrich (St. Louis, MO) unless otherwise stated.

### Separation of Human Neutrophils

Blood samples were collected in citrate from healthy individuals and from SCA (homozygous for hemoglobin S) individuals after obtaining informed consent and approval of this study by the Ethics Committee of the University of Campinas (CAAE: 36984214.1.0000.5404). Neutrophils (>95% purity, > 98% viability) were separated from peripheral blood samples using a Ficoll-Paque gradient (English and Andersen, 1974) and resuspended in RPMI medium (Vitrocell Embriolife, Campinas, Brazil) for immediate use in assays. Demographic and hematologic data for patients that participated in the study are presented in Supplemental Table 1.

### Adhesion Assays

#### Static Neutrophil Adhesion Assays.

Briefly, after specified incubations, neutrophils (2 × 10^6^ cells/ml in RPMI medium) were seeded onto 96-well plates coated with 20 µg/ml fibronectin. Cells were allowed to adhere for 30 minutes at 37°C and 5% CO_2_ (Canalli et al., 2008), after which nonadhered cells were discarded, and wells were washed thrice with PBS. Fifty microliters of RPMI was added to each well containing cells, and varying concentrations of the original cell suspension were added to empty wells to form a standard curve. Cell adhesion was calculated as a percentage by measuring the myeloperoxidase content of each well and comparing it with the appropriate standard curve.

#### Microfluidic Assays.

The Venaflux platform (Cellix Ltd., Dublin, Ireland) was used to measure neutrophil adhesion under flow conditions. Biochip (Vena8 biochips; Cellix Ltd.) microchannels (400 µm wide) were precoated (overnight, 2–8°C) with recombinant fibronectin (20 µg/ml), and nonspecific binding sites were subsequently blocked with 1% bovine serum albumin/PBS. Neutrophils (2.5 × 10^6^ cells/ml) were perfused over microchannels (initial flow rate of 3.3 nl/s; initial shear stress of 0.5 day/cm^2^) for 3 minutes. For each channel, 180 images were acquired (1/s) using a Zeiss microscope (×20 lens; Gottingen, Germany) and DeltaPix Camera (Nibe, Denmark). Neutrophil adhesion to microchannels at 3 minutes was calculated using the DucoCell analysis program (Cellix Ltd.), recording the mean number of neutrophils adhered to an area of 0.09 mm^2^.

### K562 Cell Culture and CD34^+^ Isolation

Erythroleukemic K562 cells, acquired from the American Type Culture Collection (Manassas, VA), were grown in Dulbecco’s modified Eagle’s medium supplemented with 10% fetal bovine serum, 100 U/ml penicillin, and 100 g/ml streptomycin. Cultures were incubated at 37°C in an atmosphere of 5% CO_2_ in air with extra humidity. K562 cells were incubated with agents on the first day of subculture, and the medium was not changed during the 96-hour induction period. Hydroxyurea, BAY 41-2272, and BAY 60-2770 were dissolved in minimal quantities of DMSO. All cells used for culture were in the log phase of exponential growth, and alterations in cell viability or growth phase were monitored during treatment using trypan blue exclusion assays. Control cultures were grown in the presence of the equivalent quantities of drug vehicle (DMSO or sterile water) to those used in the treated culture. CD34^+^ hematopoietic stem cells were isolated from three healthy donor volunteers according to (Almeida et al., 2008).

### Flow Cytometry

#### Neutrophils.

Isolated neutrophils (1.0 × 10^6^ cells/ml in RPMI) were incubated with allophycocyanin-conjugated mouse anti-human CD11b (clone M1/70; eBioscience) and FITC-conjugated mouse anti-human CD11a (clone HI111; eBioscience) to evaluate expressions of the CD11b (Mac-1 subunit) and CD11a (LFA-1 subunit) molecules on the neutrophil surface. For detection of activation-specific epitopes on the CD11a and CD11b molecules, cells were incubated with mouse anti-human CD11a (MEM-83; eBioscience) and FITC-conjugated rat anti-mouse IgG1 and phycoerythrin-conjugated mouse anti-human CD11b Ab (CBRM1/5; eBioscience), respectively (30 minutes, 4°C, in the dark). Ten thousand events were acquired on a FACScalibur (BD Biosciences) using the 488-nm laser and employing an forward scatter/ side scatter gate (CellQuest Software; BD Biosciences). Data are expressed as MFI and were compared with a negative isotype control using the FlowJo analysis software (Tree Star, Ashland, OR).

#### K562 Cells.

HbF protein expression was monitored in K562 (1 × 10^5^) cells after permeabilization (Fix & Perm Cell permeabilization kit; Life Technologies Corp., MD) and incubation in PBS with FITC-conjugated anti-fetal hemoglobin antibody (Life Technologies Corp.) according to the manufacturer’s instructions. Events were acquired and analyzed as described above for neutrophil flow cytometry.

### Generation of Control and SCD Chimeric Mice

Male C57BL/6 mice were obtained from the animal breeding facility at the University of Campinas, Brazil, and were housed four to five mice per cage with free access to food and water. Animals were fed on a 22% protein diet (NUVILAB CR1 irradiated) without additional arginine supplementation. Chimeric SCD mice and chimeric C57BL/6 mice were generated from the transplantation of bone marrow from Berkeley transgenic sickle cell disease mice (Tg[Hu-miniLCR1GAS] Hba/Hbb/) or C57BL/6 mice into lethally irradiated male C57BL/6 mice (6 weeks of age), as previously described (Turhan et al., 2002; Almeida et al., 2012). Only chimeric SCD mice expressing 97% human globin (phenotyped by polyacrylamide gel electrophoresis (Turhan et al., 2002)) at 3 months post-transplantation were used at 3–5 months after transplantation; these mice are hereafter referred to as SCD mice and control mice, respectively. All experimental procedures were approved by the Animal Care and Use Committee of the University of Campinas (protocols: 3121-1, 4439-1, 5358-1) and performed in accordance with the Guide for the Care and Use of Laboratory Animals as adopted and promulgated by the US National Institutes of Health. All efforts were made to minimize animal suffering and to use the minimum number of animals to produce replicable results.

### Intravital Microscopy Protocols

Inflammation was induced in mice by the injection of murine TNF (0.5 μg, i.p./200 μl) and mice were concomitantly treated with hydroxyurea (100 mg/kg, i.v.), BAY 41-2272 (10 µg/mouse), BAY 60-2770 (10 µg/mouse, i.v.), or a combination of two compounds or vehicle (2.5% v/v DMSO). Compounds were administered (either individually or in combination) in a single injection of 150 μl. At 2 hours after treatment administrations, mice were anesthetized and tracheostomized. The cremaster muscle was surgically exteriorized before continuously superfusing with bicarbonate-buffered saline (37°C, pH 7.4) and equilibrated with a 95% N_2_ and 5% CO_2_ mixture. Microvessels (6–15 for each mouse, 15–30 µm in diameter) were visualized at 3 hours after surgery using an Axio Imager D2 microscope (63× magnification; Carl Zeiss Microscopy, Jena, Germany) custom designed for intravital microscopy, and images were recorded for 30–90 seconds (40 frames/s) using an Axiocam 503 monochromatic camera (Carl Zeiss Microscopy). Rolling, adhesion, and extravasation of leukocytes (white blood cell) were monitored and analyzed for 30–45 minutes after surgery. Definitions of leukocyte rolling, adhesion, and extravasation are described in (Turhan et al., 2004). Concentrations of BAY 41-2272 and BAY60-2770 administered in vivo were determined using drug response dosing experiments in C57BL/6 mice (Supplemental Fig. 1) and based on previous studies (Wang et al., 2013; Ghosh et al., 2016). The hydroxyurea dose employed was based on previous in vivo studies from our groups (Almeida et al., 2012, 2015).

### Quantification of Plasma cGMP

Blood was collected from mice within 5 hours of administration, or not, of the drugs studied. Plasma was separated from the blood (2500*g* for 10 minutes), and samples were then stored at −80°C. Plasma cGMP was quantified using commercially available ELISA kits (GE Healthcare, Chicago, IL).

### Quantitative Real-Time Polymerase Chain Reaction and Gene Expression Analysis

Total RNA was extracted from cells of interest using Trizol reagent or an RNA extraction kit (RNeasy mini kit; Qiagen, Hilden, Germany) according to the manufacturers’ protocols. cDNA was synthesized from total RNA extracts with RevertAid H minus First Strand cDNA synthesis kit (ThermoFisher Scientific, MA). Synthetic oligonucleotide primers were designed to amplify cDNA for genes encoding *γ*-globin (*HBG*), soluble guanylate cyclase *α* subunit (*GUCY1A1*), soluble guanylate cyclase *β* subunit (*GUCY1B1*), and *β*-actin (*ACTB*) and glyceraldehyde phosphate dehydrogenase (*GAPDH*) (Primer Express; Applied Biosystems, Foster City, CA). Primers were synthesized by Integrated DNA Technologies (Coralville, IA); for *HBG*, *ACTB*, and *GAPDH* primer sequences and concentrations, see Dos Santos et al. (2012). Primer sequences for amplifying *GUCY1A1* were as follows: forward, 5′-ATG​CAC​TGT​ACA​CTC​GCT​TCG​A-3′ and reverse, 5′- CAA​CGA​CGC​CAG​CAA​AAA​C-3′. Primer sequences for *GUCY1B1* were as follows: forward, 5′- GCC​AGG​TTC​AAG​TAG​ATG​GTG-3′ and reverse, 5′- GGC​ATC​CGC​TGT​CCT​ATG-3′. All samples were assayed in a 12-μl volume containing 10 ng of cDNA (3.0 μl), 6.0 μl of SYBR Green Master Mix PCR (Applied Biosystems), and 3.0 μl of specific primers in a MicroAmp Optical 96-well reaction plate (Applied Biosystems) using the StepOne Plus (Applied Biosystems), as previously described. Gene expression was quantified using the Gnorm program. Two replicas were run on the plate for each sample. For *HBG* gene expression, results are expressed as mRNA levels normalized according to the expressions of *ACTB* and *GAPDH*. For the *GUCY1A1/GUCY1B1* genes, relative expression was calculated as the fold change in mRNA quantity according to the 2(−ΔΔCt) method and normalized to the expression of *ACTB*.

### Statistical Analysis

Values are expressed as means ± S.E.M. Data were confirmed as parametric or not, and comparisons were performed using ANOVA with multiple comparisons post-test as appropriate and as specified. Differences among groups were considered significant at *P* ≤ 0.05.

## Results

### 

#### sGC Stimulation and sGC Activation Inhibit Ex Vivo SCA Human Neutrophil Adhesive Mechanisms.

**Fig. 1.**
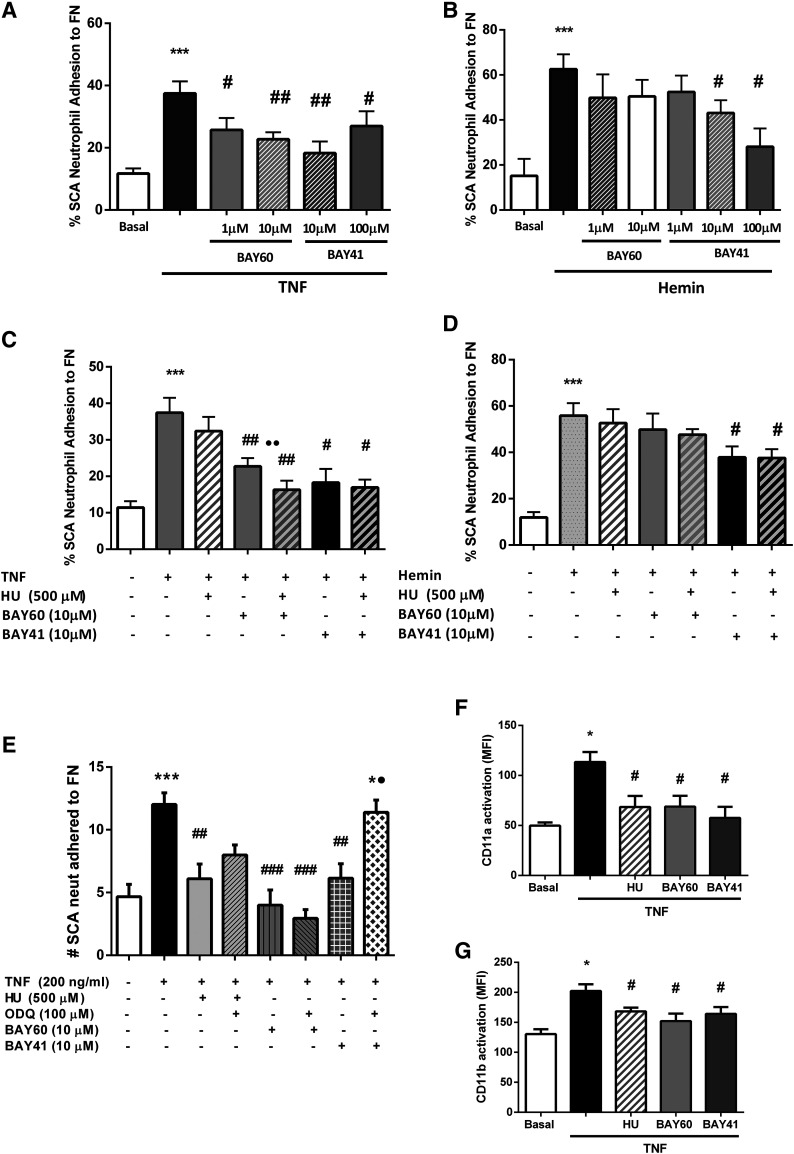
Effects of BAY 60-2770 and BAY 41-2272 on the adhesion of neutrophils from individuals with SCA to fibronectin ligand. Static adhesion assays: Neutrophils isolated from individuals with SCA (*N* = 5–12) were incubated for 90 minutes with BAY 60-2770 (BAY60) or BAY 41-2272 (BAY41) before stimulating with (or without) (A) TNF cytokine (200 ng/ml, 30 minutes coincubation) or (B) hemin (50 µM, 30 minutes coincubation) and observing adhesion of neutrophils to fibronectin (FN) ligand (30 minutes, 37°C, 5% CO_2_). Subsequently, the effects of the coincubation of cells with HU (500 µM, 15 minutes) on (C) TNF cytokine–induced (200 ng/ml, 30 minutes) and (D) hemin-induced (50 µM, 30 minutes) adhesion of SCA neutrophils (*N* = 5–12) to fibronectin ligand (30 minutes, 37°C, 5% CO_2_) were determined. (E) Microfluidic adhesion assay: Adhesion of neutrophils from individuals with SCA (*N* = 6 to 7) to fibronectin ligand in 400-μm-wide channel biochips using a shear rate of 0.5 dynes/cm^2^ (3 minutes, 37°C) was determined after coincubating cells with HU (500 µM, 45 minutes), BAY 41-2272 (90 minutes), or BAY 60-2770 (90 minutes) in the presence or absence of the sGC inhibitor ODQ (100 µM) before costimulating or not with TNF cytokine (200 ng/ml, 30 minutes, 37°C); neutrophils adhered to fibronectin represent the number of neutrophils adhered in a field of 0.09 mm^2^. *P < 0.05; ***P < 0.001, compared with basal adhesion. ^#^*P* < 0.05; ^##^*P* < 0.01; ^###^*P* < 0.001, compared with TNF-stimulated or hemin-stimulated adhesion. ●*P* < 0.05; ●●*P* < 0.01, compared with BAY60 or BAY41 alone (as appropriate; ANOVA and Sidak’s multiple comparison test). Effects of BAY 60-2770 and BAY 41-2272 on the activation conformation of the CD11a (F) and CD11b (G) integrin subunits on neutrophils isolated from healthy subjects. Cells were incubated with HU (100 µM), BAY 41-2272 (10 µM), or BAY 60-2770 (10 µM) for 30 minutes (37°C, 5% CO_2_) before the addition, or not, of TNF (200 ng/ml; 30 minutes). Flow cytometry was employed to compare antibody binding to the activated epitopes of CD11a and CD11b (antibody clones MEM-83 and CBRM1/5, respectively). Integrin subunit activation is shown as the mean ± S.E.M.; *N* = 6–8 for each group. *P < 0.05, compared with basal. #*P* < 0.05, compared with TNF alone (ANOVA and Sidak’s multiple comparison test).

As previously reported (Canalli et al., 2008; Miguel et al., 2011), neutrophils isolated from individuals with SCA often demonstrate an increased capacity to adhere to fibronectin ligand in vitro when compared with neutrophils from healthy individuals without SCA. Given that stimulation of cGMP-dependent signaling has demonstrated beneficial effects on vaso-occlusive mechanisms (Almeida et al., 2012), we first semiquantified the expressions of the genes encoding the sGC *α* and *β* subunits in neutrophils from healthy individuals (Supplemental Fig. 2). As gene expression of both sGC subunits was confirmed in these cells, albeit at lower levels than those observed in CD34^+^ hematopoietic stem cells, we then looked at how sGC agonism may modulate neutrophil adhesive properties. Neutrophils from individuals with SCA were then incubated with either BAY 60-2770 or BAY 41-2272 (90 minutes) before costimulating with recombinant TNF cytokine (200 ng/ml, 30 minutes) or hemin (50 µM, 30 minutes) and determining cellular adhesion to fibronectin using static adhesion assays (30 minutes, 37°C, 5% CO_2_). TNF and hemin each augmented the adhesion of SCA neutrophils (Fig. 1, A and B). Both BAY 60-2770 (1–10 µM) and BAY 41-2272 (10–100 µM) significantly decreased the TNF-stimulated adhesion of SCA neutrophils to fibronectin (Fig. 1A), whereas only BAY 41-2272—at concentrations of 10 and 100 µM, but not 1 µM—significantly inhibited hemin-induced SCA adhesion to fibronectin (Fig. 1B). With regard to neutrophils from healthy control individuals, neither BAY 41-2272 nor BAY 60-2770, at the same concentrations, significantly inhibited either TNF- or hemin-induced healthy control neutrophil static adhesion to fibronectin (Supplemental Fig. 3, A and B).

The inhibiting effects of the sGC activator/stimulator on TNF-stimulated SCA neutrophil adhesion to fibronectin were confirmed using microfluidic assays, which afforded cell flow through biochip channels of similar widths to those of small blood vessels (400 µm) utilizing a shear stress of 0.5 dynes/cm^2^ for 3 minutes. Under conditions of flow, the adhesion of neutrophils from individuals with SCA was significantly inhibited by BAY 60-2770 and BAY 41-2272 (10 µM; Fig. 1E). In contrast, neither BAY 41-2272 nor BAY 60-2770 at the concentrations employed significantly inhibited TNF-stimulated adhesion of healthy control neutrophils in the microfluidic assay (Supplemental Fig. 3E).

Cell viability assays (3-(4,5-dimethylthiazol-2-yl)-2,5-diphenyltetrazolium bromide, MTT) demonstrated that neither BAY 60-2770 (1–10 µM) nor BAY 41-2272 (1–100 µM) affected neutrophil viability (data not shown) at the concentrations depicted.

#### Hydroxyurea Potentiates the Effects of sGC Activation on Ex Vivo Human Neutrophil Adhesion to Fibronectin.

Hydroxyurea is suggested to have NO donor properties. To investigate the influence of hydroxyurea on the effects of sGC stimulation and activation, SCA neutrophils were coincubated with BAY 60-2770 or BAY 41-2272 (10 µM; 90 minutes) and hydroxyurea (500 µM, 15 minutes) before observing their adhesion to fibronectin under static conditions (30 minutes, 37°C, 5% CO_2_). Under the static conditions used, hydroxyurea alone did not significantly inhibit either TNF- or hemin-induced SCA neutrophil adhesion to fibronectin (Fig. 1, C and D). Cotreatment of TNF-stimulated SCA neutrophils with BAY 60-2770 (10 µM, 90 minutes) together with hydroxyurea further decreased their adhesion to fibronectin but did not significantly alter the effect of BAY 41-2272 (10 µM; 90 minutes) on TNF-stimulated SCA neutrophil adhesion (Fig. 1C). In contrast, coincubation of SCA neutrophils with hydroxyurea did not modulate the effects of either of the compounds on hemin-induced neutrophil adhesion to fibronectin (Fig. 1D; *P* > 0.05).

When TNF-stimulated neutrophils from healthy control individuals were coincubated with BAY 60-2770 (10 µM) together with hydroxyurea (500 µM, 15 minutes), a significant inhibition of cell adhesion to fibronectin occurred (Supplemental Fig. 3C); however, no potentiation of the effects of BAY 41-2272 (100 µM) on either TNF- or hemin-induced healthy control neutrophil adhesion was observed (Supplemental Fig. 3, C and D).

#### The Inhibiting Effects of BAY 41-2272, but not BAY 60-2770, on TNF-Induced SCA Neutrophil Adhesion are Reversed by sGC Oxidation.

The effect of the oxidation of the heme moiety of sGC on the ability of hydroxyurea, BAY41-2272, and BAY 60-2770 to inhibit TNF-induced SCA neutrophil adhesion to fibronectin was evaluated using microfluidic assays. Neutrophils were pretreated with either hydroxyurea (500 µM, 45 minutes), BAY 60-2770, or BAY 41-2272 (10 µM, 90 minutes) in the presence or absence of ODQ (100 µM; sGC oxidant) and costimulated with TNF (30 minutes) before perfusing over fibronectin-coated channels (Fig. 1E). Hydroxyurea significantly decreased TNF-induced adhesion to fibronectin under microfluidic conditions; however, the slight reversal of this effect by ODQ was not statistically significant (*P* > 0.05). Expectedly, ODQ was able to reverse the effect of BAY 41-2272 on SCA neutrophil adhesion (Fig. 1E), consistent with the mechanism of action of sGC stimulators, whereas sGC oxidation did not reverse the inhibiting effect of BAY 60-2770 on SCA neutrophil adhesion in response to TNF stimulation.

Since significant inhibition of TNF-stimulated healthy control neutrophil adhesion to fibronectin after BAY 60-2770 or BAY 41-2272 (10 µM, 90 minutes) preincubation was not observed under flow conditions, coincubation with ODQ had no significant effects on these mechanisms (Supplemental Fig. 3E).

#### sGC Activation and sGC Stimulation Inhibits TNF-Induced Mac-1 and LFA-1 Integrin Activation.

Neutrophil adhesive interactions with the extracellular matrix and endothelium are largely mediated by the actions of the LFA-1 (CD11a/CD18) and MAC-1 (CD11b/CD18) integrins on the cell surface. The expressions of the CD11a and CD11b subunits on the surface of healthy control neutrophils after incubation of the cells with TNF cytokine (200 ng/ml, 30 minutes) and after preincubation (30 minutes) or not with hydroxyurea (100 µM), BAY 41-2272 (10 µM), or BAY 60-2770 (10 µM) were determined by flow cytometry. The activation states of these integrin subunits were also determined by using activation epitope-specific antibodies.

Although TNF did not significantly modulate CD11a and CD11b surface expression (Supplemental Table 2) on healthy control neutrophils under the conditions used, this cytokine significantly augmented the binding activities of these integrin subunits on neutrophils (Fig. 1, F and G). In turn, similarly to hydroxyurea, both BAY 41-2272 and BAY 60-2770 significantly abrogated this TNF-induced increase in LFA-1 and Mac-1 integrin activity (Fig. 1, F and G).

#### sGC Activation and sGC Stimulation Reduce Leukocyte Recruitment in the Microvasculature of TNF-Stimulated Chimeric SCD Mice.

TNF-induced leukocyte recruitment to the microvascular wall triggers vaso-occlusive mechanisms in mice with SCD. To evaluate the effects of intracellular cGMP modulation on leukocyte recruitment, we administered hydroxyurea (100 mg/kg, i.v.), BAY 60-2770 (10 µg/mouse, i.v.), and/or BAY 41-2272 (10 µg/mouse, i.v.) to chimeric SCD mice immediately before inducing leukocyte recruitment with TNF (0.5 μg, i.p.). Leukocyte recruitment to the microvasculature of the cremaster muscle was then observed at 180 minutes post–TNF administration by intravital microscopy.

**Fig. 2.**
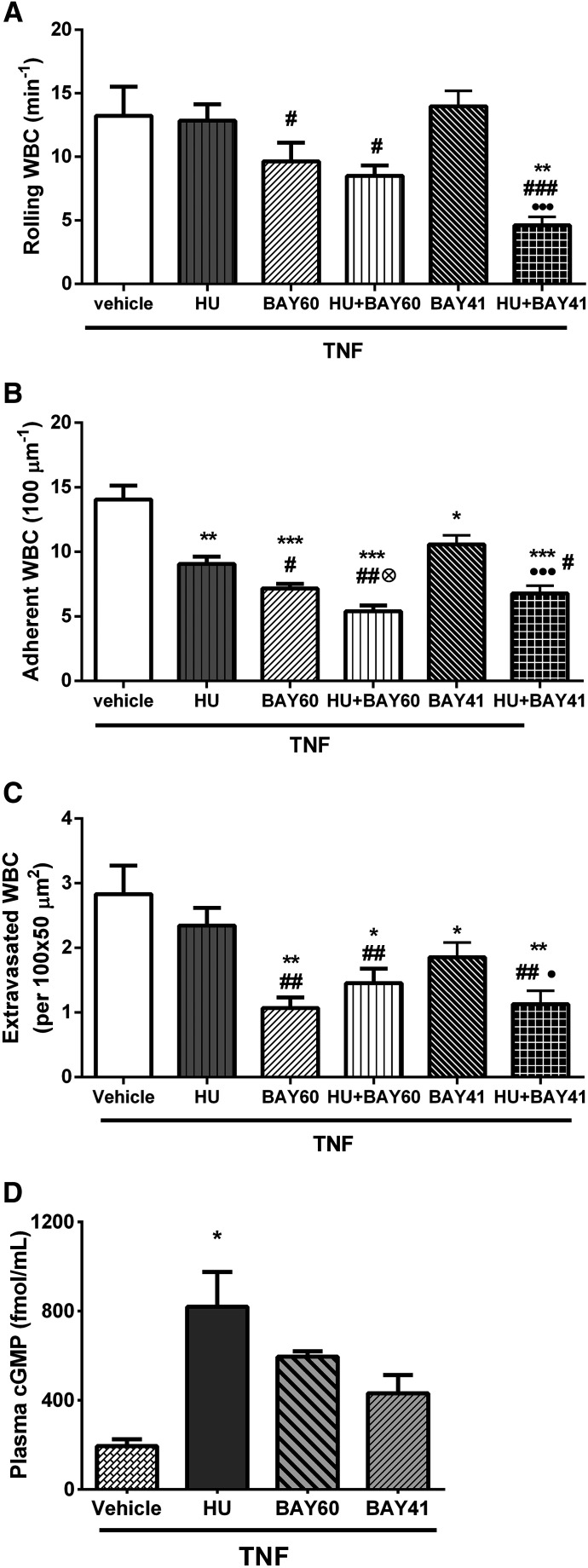
BAY 60-2770 and BAY 41-2272 inhibit leukocyte recruitment to the microvasculature in a murine model of SCD inflammatory vaso-occlusion. Vaso-occlusive–like processes were induced in SCD mice by administration of TNF (0.5 µg, i.p.). Mice concomitantly received, or not, administrations of saline vehicle, HU (100 mg/kg), BAY 60-2770 (10 µg/mouse), and/or BAY 41-2272 (10 µg/mouse). (A) Leukocyte rolling, (B) leukocyte adhesion, and (C) leukocyte extravasation were quantified in venules of SCD mice (four to six mice per group; 6–10 venules per mouse) at 180 minutes after the administration of TNF. **P < 0.01; ***P < 0.001, compared with TNF alone. ^#^*P* < 0.05; ^##^*P* < 0.01; ^###^*P* < 0.001, compared with HU alone. ⊗*P* < 0.05, compared with BAY 60-2770 alone. ● *P* < 0.05; ●●●*P* < 0.001, compared with BAY 41-2272 alone. (D) Measurement of plasma cGMP in SCD mice after administration of TNF (0.5 µg, i.p., 180 minutes) concomitantly with saline vehicle or HU (100 mg/kg), BAY 41-2272 (BAY41, 10 µg/mouse), or BAY 60-2770 (BAY60, 10 µg/mouse). Plasma cGMP was measured by ELISA. *N* = 3–7 mice per group; *P < 0.05, compared with TNF alone (ANOVA and Dunn’s multiple comparison test). WBC, white blood cell.

As previously demonstrated, and under the conditions employed, administration of a single dose of hydroxyurea modulated leukocyte recruitment in the microvasculature, significantly reducing the leukocyte adhesion and extravasation induced by TNF without significant alterations in rolling activity (Fig. 2, A–C; Supplemental Fig. 4). The effects of BAY 60-2770 or BAY 41-2272 at the concentrations used were very similar to those of hydroxyurea, although BAY 41-2272 apparently inhibited leukocyte recruitment less efficiently (*P* > 0.05) than BAY 60-2770 (Fig. 2, A–C; Supplemental Fig. 4). Importantly, coadministration of hydroxyurea together with either of the sGC modulators significantly potentiated their effects on leukocyte recruitment, reducing leukocyte rolling and further decreasing cell adhesion and extravasation in relation to either hydroxyurea alone or the agonists alone (Fig. 2, A–C).

In control mice, the administration of hydroxyurea (100 mg/kg, i.v.) and BAY 41-2272 (10 µg/mouse, i.v.) also significantly decreased TNF-induced leukocyte adhesion and extravasation (Supplemental Fig. 5, B and C) in the microvasculature in association with an elevation in leukocyte rolling (Supplemental Fig. 5A). In contrast, BAY 60-2770 (10 µg/mouse, i.v.) significantly abrogated TNF-induced rolling, adhesion, and extravasation (Supplemental Fig. 5, A–D).

#### Effects of BAY 41-2272 and BAY 60-2770 on Plasma cGMP in Chimeric SCD Mice.

To look at the ability of the sGC stimulator/activators to modulate cGMP levels in SCD mice when administered immediately before TNF stimulation (0.5 μg, i.p.), we collected plasma from mice within 5 hours of administration. Figure 2D demonstrates that a single dose of hydroxyurea (100 mg/kg, i.v.) significantly increased plasma cGMP. Augmentations in plasma cGMP after BAY 60-2770 and BAY 41-2272 (10 µg/mouse, i.v.) were not found to be statistically significant. Hydroxyurea (100 mg/kg, i.v.), BAY 60-2770, and BAY 41-2272 (10 µg/mouse, i.v.) also elevated plasma cGMP in control mice (Supplemental Fig. 5E), but this increase was only significant for BAY 60-2770–treated mice.

#### BAY 41-2272 Induces Gene Expression of *γ*-Globin and Fetal Hemoglobin Protein Production in Erythroid Lineage Cells.

**Fig. 3.**
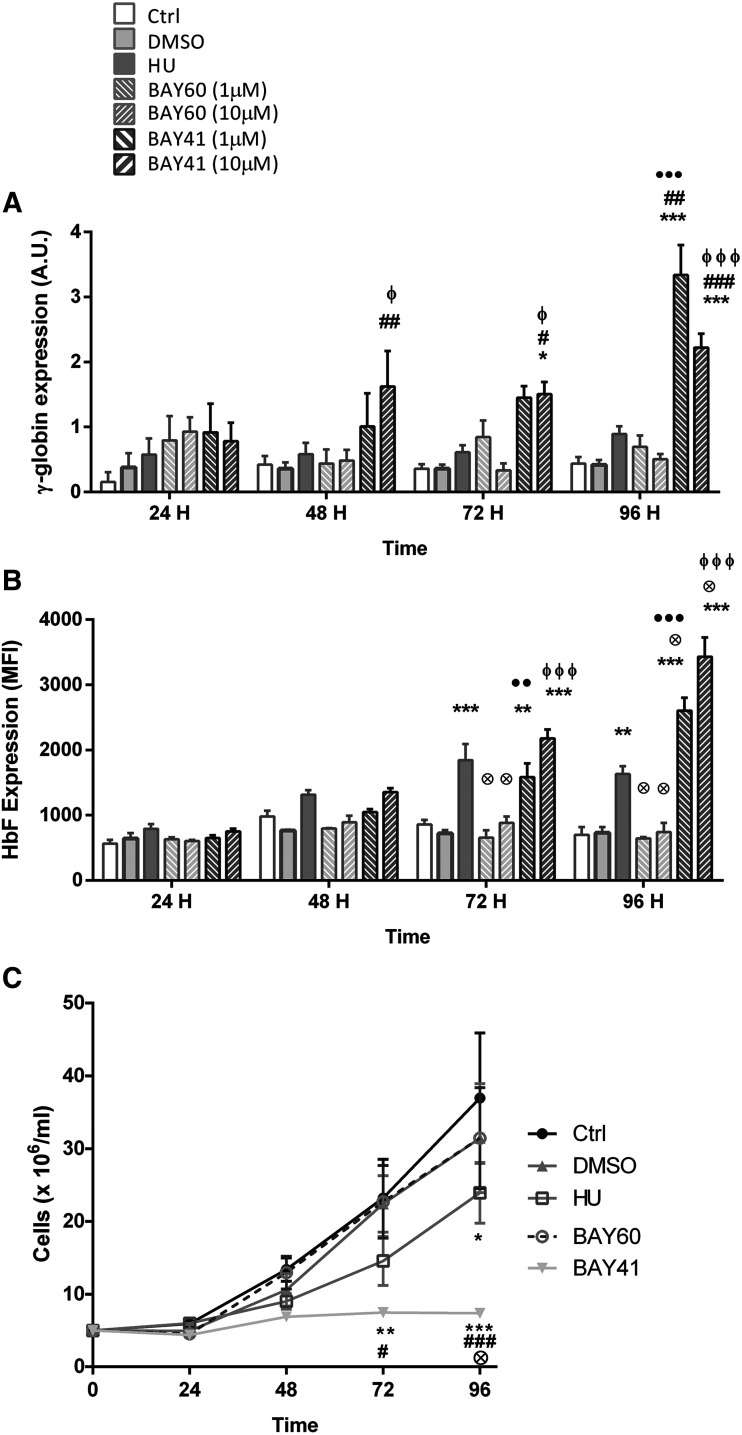
Effects of soluble guanylyl cyclase activity modulation on *γ*-globin and HbF expression in K562 erythroleukemic cells in culture. Cells were coincubated with HU (100 µM), BAY 60-2770 (1–10 µM), BAY 41-2272 (1–10 µM), or DMSO vehicle (0.1% v/v) for 96 hours (37°C, 5% CO_2_). (A) *HBG* gene expression was determined by quantitative real-time polymerase chain reaction normalized to *ACTB* and *GAPDH* expression; (B) HbF was determined by flow cytometry, and (C) cell proliferation was determined (BAY 60-2770 and BAY 41-2272, 10 µM). (A) *P < 0.05; **P < 0.01; ***P < 0.001, compared with untreated cells (Ctrl). ^#^*P* < 0.05; ^##^*P* < 0.01; ^###^*P* < 0.001, compared with DMSO alone. ●●●*P* < 0.001, compared with BAY 60-2770 (1 µM). ɸ ɸ ɸ, compared with BAY 60-2770 (10 µM). (B) **P < 0.01; ***P < 0.001, compared with Ctrl and DMSO. ⊗*P* < 0.05, compared with HU. ●●*P* < 0.01; ●●●*P* < 0.001, compared with BAY 60-2770 (1 µM). ɸ ɸ ɸ, compared with BAY 60-2770 (10 µM). (C) *P < 0.05; **P < 0.01; ***P < 0.001, compared with Ctrl. ^#^*P* < 0.05; ^###^*P* < 0.001, compared with DMSO alone and BAY 60-2770 (10 µM). ⊕*P* < 0.01, compared with HU. Two-way ANOVA, Tukey’s multiple comparisons test.

Given the fact that drugs with HbF-elevating properties are highly beneficial in SCA and that evidence suggests a role for cGMP-dependent signaling in *γ*-globin regulation, we looked at the ability of BAY 41-2272 and BAY 60-2770 to increase the expression of the gene encoding *γ*-globin, *HBG*, in the erythroleukemic cell line K562. Having confirmed the expressions of the genes encoding the sGC subunits in K562 cells (Supplemental Fig. 2), these cells were cultured in the presence or absence of hydroxyurea (100 µM), BAY 60-2770 (10 µM), BAY 41-2272 (10 µM), or DMSO vehicle (0.1% v/v) for 96 hours. *HBG* expression in cells was determined by quantitative real-time polymerase chain reaction and HbF protein expression was measured by flow cytometry (Fig. 3).

As expected, hydroxyurea increased *HBG* expression in the K562 cells by approximately 2-fold at 48–96 hours of culture (Fig. 3A) in association with a significant increase in HbF protein expression at 72–96 hours (Fig. 3B). Surprisingly, coculture of the cells with BAY 41-2272 triggered *HBG* expression and HbF production much more efficiently than hydroxyurea at all time points, augmenting HbF expression by more than 3-fold by 96 hours of culture. In contrast, BAY 60-2770 at the concentrations employed (data for 1 and 100 µM BAY 60-2770 not shown) did not show such an upregulating effect on *HBG* expression and, consequently, HbF production in the erythroleukemic cells.

Interestingly, the effects of hydroxyurea and BAY 41-2272 on HbF production were both accompanied by inhibition of the proliferation (but not induction of cell death) of the K562 cells (Fig. 3C), consistent with the known cytostatic property of hydroxyurea.

## Discussion

Sickle cell anemia is now recognized as a global health problem (Kato et al., 2018), and although curative therapy for SCA exists in the form of hematopoietic stem cell transplantation, its availability is very restricted (Yawn et al., 2014). Despite concerted efforts by researchers and the pharmaceutical industry to develop novel drugs for the treatment of SCA (Telen, 2016; Conran and Torres, 2019), only three substances have been approved by the Food and Drug Administration for SCA therapy since hydroxyurea’s approval over 20 years ago, and it is probable that multidrug approaches, possibly still involving the use of hydroxyurea, will evolve for the management of the disease (Carden and Little, 2019). Recruitment of leukocytes to the vascular wall constitutes a major trigger for vaso-occlusion in SCA and occurs in response to inflammatory stimuli such as TNF cytokine, cell-free heme (or hemin, a damage-associated molecular pattern), and ischemia/reperfusion processes (Turhan et al., 2002; Kalambur et al., 2004; Belcher et al., 2014). As such, inhibiting the adhesion of leukocytes to the vascular endothelium under inflammatory conditions may represent an important approach for preventing SCA vaso-occlusion (Chang et al., 2010) and its consequent complications.

Neutrophils from individuals with SCA generally demonstrate augmented adhesive properties compared with neutrophils from healthy individuals, and this adhesion is further augmented by cellular activation by the inflammatory molecules TNF cytokine and hemin, both of which are elevated in the circulation of individuals with SCA (Lanaro et al., 2009; Schaer et al., 2013). Neutrophils express the genes encoding sGC, albeit at significantly lower levels than those encountered in CD34^+^ hematopoietic cells. As stimulation of cGMP-dependent signaling may have beneficial consequences on leukocyte function (Almeida et al., 2012; Conran and Torres, 2019) in SCA, we investigated the effects of the sGC activator and sGC stimulator, BAY 60-2770 and BAY 41-2272, respectively, on the ex vivo adhesive properties of neutrophils from patients with SCA. BAY 60-2770 at relatively low in vitro concentrations (1–10 µM) effectively inhibited the adhesion of SCA neutrophils to fibronectin after TNF-induced activation but not hemin-induced SCA neutrophil adhesion. Notably, BAY 41-2272 (from 10 µM) significantly inhibited the adhesive properties of SCA neutrophils when activated by hemin in addition to diminishing TNF-induced SCA neutrophil adhesion. BAY 41-2272 and BAY 60-2770 are preclinical tool compounds and have not been tested in patients to allow direct comparison of exposure and pharmacokinetics with the in vitro doses employed (Zenzmaier et al., 2015), but concentrations of these agonists of 1–30 µM are consistently employed in in vitro studies (Zenzmaier et al., 2015). Consistent with the sGC activating and stimulating abilities of BAY 60-2770 and BAY 41-2272 (Follmann et al., 2013), respectively, oxidation of sGC by ODQ slightly potentiated the inhibiting effect of BAY 60-2770 on neutrophil adhesion while reversing the effect of BAY 41-2272.

Flow cytometry assays suggest that the observed effects of both sGC agonists on TNF-induced neutrophil adhesion appear to be mediated by the suppression of the activation of the Mac-1 and LFA-1 integrins on the neutrophil cell surface, which is in keeping with previous reports of a role for the NO/cGMP pathway in regulating leukocyte integrin function (Conran et al., 2001, 2003). In vivo, BAY 60-2770 and BAY 41-2272, when administered alone, significantly diminished leukocyte recruitment to the TNF-stimulated microvasculature of SCD mice (as demonstrated by decreased leukocyte adhesion and extravasation at the microvessel walls). Of note, although the in vitro effects of BAY 41-2272 on neutrophil adhesive properties appeared to be superior, the effects of BAY 60-2770 in vivo were apparently slightly more efficient under the conditions employed.

Hydroxyurea has previously been shown to inhibit leukocyte recruitment in SCD mice after a single administration and via a mechanism mediated by cGMP-dependent signaling (Almeida et al., 2012). In vitro, coincubation of SCA neutrophils with hydroxyurea potentiated the effects of BAY 60-2770 on their adhesion. Given that BAY 60-2770 activates oxidized sGC in an NO-independent manner (Pankey et al., 2011), the amplification of its effect by hydroxyurea was somewhat surprising. Furthermore, in vivo, the concomitant administration of hydroxyurea to SCD mice amplified the effects of both BAY 60-2770 and BAY 41-2272 on leukocyte recruitment in the microvasculature, further inhibiting leukocyte adhesion to blood vessel walls when used in combination with a sGC activator/stimulator. The beneficial effects of BAY 60-2770 and BAY 41-2272 in the microvasculature of mice with SCD were associated with improvements in circulating cGMP levels. As such, the potentiation of the effects of BAY 60-2770 by hydroxyurea suggests that, in this model, hydroxyurea may in fact modulate sGC activity in conjunction with the sGC activator, rather than provide NO supplementation. Another explanation could be that constant shifts can occur in the redox equilibrium, modulating the balance of oxidized and native sGC and, therefore, the sensitivity of this enzyme to NO (Breitenstein et al., 2017). However, to confirm these notions, further investigations are necessary.

As mentioned, a major effect of hydroxyurea is its ability to induce HbF (fetal hemoglobin, *α*2*γ*2) production in erythroid cells, in turn reducing sickle hemoglobin polymerization and red cell sickling (Platt et al., 1984). Drugs in development for the treatment of SCA should ideally be able to elevate HbF levels if their use is proposed to replace that of hydroxyurea in a chronic regimen. In fact, activation of the sGC-cGMP-dependent protein kinase pathway can upregulate the expression of the *γ*-globin gene, in turn increasing HbF generation (Ikuta et al., 2001); furthermore, there is evidence that the HbF-inducing ability of hydroxyurea in erythroid cells could well be mediated by inducing sGC activity (Cokic et al., 2003, 2008). As such, we compared the abilities of BAY 60-2770 and BAY 41-2272 with that of hydroxyurea to induce *γ*-globin (*HBG*) gene transcription and the expression of HbF protein in an erythroleukemic cell line. Hydroxyurea (100 µM) significantly induced *HBG* gene expression within 48 hours of culture in association with increased HbF generation after 72 hours. sGC stimulation with BAY 41-2272 (10 µM) significantly increased both *HBG* gene and HbF protein expression within a similar time frame and apparently even more efficiently than hydroxyurea. Like hydroxyurea, BAY 41-2272 decreased erythroid cell proliferation without inducing cell death, indicating that BAY 41-2272 may mediate this effect via a cytostatic action. In contrast, BAY 60-2770 at the concentrations employed (1–100 µM) induced neither *HBG* gene nor HbF protein expression. Given the similar, or even slightly superior, effects of the sGC activator in vivo, the effects of these agonists on *HBG* gene and HbF protein expression should be further investigated.

One limitation of this study is that the intravenous effects of both sGC agonists on murine blood pressure were not assessed. However, it is well established that sGC stimulators and sGC activators have dose-dependent blood pressure–lowering effects. Moreover, intravenous doses of 100 µg/kg of BAY 41-2272 significantly decrease blood pressure in anesthetized rats (Straub et al., 2002), whereas in conscious rats, both BAY412272 and BAY60-2770, when given intravenously (300 µg/kg), are reported to have no significant effect on blood pressure (Fullhase et al., 2015). Olinciguat, another sGC stimulator, which is currently in phase 2 clinical development for use in patients with sickle cell anemia (NCT03285178) (Zimmer et al., 2020), not only reduces blood pressure in humans and in hypertensive and normotensive rats but also successfully reduces inflammatory mechanisms in TNF-stimulated mice (Buys et al., 2018; Zimmer et al., 2020). As such, further preclinical and clinical studies should be careful to evaluate the extent of any effect of sGC agonists on blood pressure when considering the use of these agents for the treatment of SCA.

In conclusion, this study suggests that both sGC activation and stimulation could represent approaches to reduce leukocyte recruitment to the endothelium and, therefore, reduce vaso-occlusive episodes in SCD. Although sGC activation could potentially reduce leukocyte recruitment without concomitant hydroxyurea administration, this class of drugs could be of use for reducing vaso-occlusive episodes in patients not on hydroxyurea therapy. Alternatively, if both HbF induction and abrogation of vaso-occlusive events are a goal for therapy, combination therapy with both sGC stimulators and hydroxyurea could offer a potent approach for SCD management.

## Abbreviations

ACTB: *β*-actin
FITC: fluorescein isothiocyanate
GAPDH: glyceraldehyde phosphate dehydrogenase
*GUCY1A1*: soluble guanylate cyclase *α* subunit
*GUCY1B1*: soluble guanylate cyclase *β* subunit
HbF: fetal hemoglobin
*HBG*: hemoglobin subunit gamma 1
HU: hydroxyurea
LFA-1: lymphocyte function-associated antigen 1
Mac-1: macrophage-1 antigen
MFI: mean fluorescence intensity
NO: nitric oxide
ODQ: 1H-[1,2,4]oxadiazolo[4,3-a]quinoxalin-1-one
SCA: sickle cell anemia
SCD: sickle cell disease
sGC: soluble guanylyl cyclase
TNF: tumor necrosis factor-*α*
